# Hypothalamic miR-204 Induces Alteration of Heart Electrophysiology and Neurogenic Hypertension by Regulating the Sympathetic Nerve Activity: Potential Role of Microbiota

**DOI:** 10.7759/cureus.18783

**Published:** 2021-10-14

**Authors:** Adam Kassan, Karima Ait-Aissa, Modar Kassan

**Affiliations:** 1 School of Pharmacy, West Coast University, Los Angeles, USA; 2 College of Medicine, University of Iowa, Iowa City, USA; 3 Physiology, The University of Tennessee Health Science Center, Memphis, USA

**Keywords:** cardiac arrhythmia, high blood pressure, cardiac electrophysiology, hypothalamic mir-204, sympathetic nerve activity

## Abstract

There is abundant evidence demonstrating the association between gut dysbiosis and neurogenic diseases such as hypertension. A common characteristic of resistant hypertension is the chronic elevation in sympathetic nervous system (SNS) activity accompanied by increased release of norepinephrine (NE), indicating a neurogenic component that contributes to the development of hypertension. Factors that modulate the sympathetic tone to the cardiovascular system in hypertensive patients are still poorly understood. Research has identified an interaction between the brain and the gut, and this interaction plays a possible role in the mechanism of heart damage-induced hypertension. Data, however, remain scarce, and further study is required to define the role of microbiota in sympathetic neural function and its relationship with heart damage and blood pressure (BP) control. Experimental evidence has pointed toward a bidirectional relationship between alterations in the types of bacteria present in the gut and neurogenic diseases, such as hypertension. Our published data showed that miR-204, a microRNA that plays an important role in the CNS function, is affected by gut dysbiosis. Therefore, miR-204 could be a key element that regulates normal sinus rhythm and neuronal hypertension. In this review, we will shed light on the potential mechanism by which microbiota affects hypothalamic miR-204, which in turn, could hinder the sympathetic nerve drive to the cardiovascular system leading to arrhythmia and hypertension.

## Introduction and background

The most common form of hypertension is neurogenic hypertension, which is defined as high blood pressure (BP) with sympathetic overdrive along with raised vasomotor tone and increased cardiac afterload [1]. Recently, the sympathetic nervous system (SNS) has gained a lot of attention in hypertension. For instance, it has been shown that sympathetic abnormalities can influence the development and progression of cardiovascular damage. Furthermore, recent studies are demonstrating that sympathetic activation has an adverse prognostic effect in terms of morbidity and mortality on a variety of cardiovascular diseases [2]. This underlines the importance of modulating sympathetic activation as a goal for non-pharmacological as well as pharmacological interventions aimed at lowering elevated BP and decreasing heart damage.

## Review

Previous research has established a bidirectional relationship between alterations in the types of bacteria present in the gut and neurogenic diseases such as hypertension and arrhythmia [3-6]. As well, several studies have shown that fecal microbiota transplantation (FMT) from hypertensive human and rat donors elevates the BP of recipient normotensive mice and rats, respectively, pointing out that gut dysbiosis plays a possible contributing role in hypertension [7-9]. Other studies also reported a close relationship between gut microbiota dysbiosis and the pathogenesis of arrhythmia [5, 10]. Data, however, remain scarce, and further investigation is required to define the role of gut dysbiosis on sympathetic function and its involvement in BP control and normal heart rate.

Recent literature shows that microbiota can regulate microRNAs (miRNAs) in the brain [3]. However, the exact mechanism through which the gut microbiota influences the expression of miRNAs remains unclear. miRNAs, a class of small, non-coding RNAs that target complementary messenger RNAs to either destabilize their structure or repress protein translation [11], regulate many cellular processes and human diseases such as hypertension, cancer, diabetes, arrhythmia, heart failure, and obesity [12]. MiR-204 is robustly expressed in many regions of the CNS [13, 14]. The absence of commensal bacteria results in the downregulation of miR-204 in the amygdala [15]. Moreover, germ-free conditions induce a transcriptional profile in the amygdala consistent with upregulation in neural activity and synaptic transmission [15]. Given the relationship between gut microbiota, the CNS, and miR-204 on one hand, and the importance of neuronal signals in regulating hypertension and heart rate on the other hand, we decided in this review to discuss whether miR-204 in the CNS regulates sympathetic nerve activity (SNA) and therefore functions as a molecular relay that converts gut bacterial signals into a neural output that affects cardiac electrophysiology and hypertension. Understanding the mechanism by which microbiota and hypothalamic miR-204 mediates SNA, heart function, and hypertension may lead to novel treatment strategies conferring the benefits of SNA control while also circumventing the potential risks of delivering live biologics.

Hypothalamic miR-204 and sympathetic nerve output

Increased sympathetic activity is a key factor in the development of hypertension and a major contributor to cardiovascular diseases [16, 17]. Neural control of BP is mediated by a core network of hypothalamic and brain stem nuclei [18]. The paraventricular nucleus (PVN) of the hypothalamus plays an important role in regulating SNA [19]. Recent studies showed that increased levels of the brain-derived neurotrophic factor (BDNF) in the PVN led to increased sympathetic activity, BP, and arrhythmia [20-23]. Interestingly, miR-204 is highly expressed in the hypothalamus. Recent research has shown that decreased level of miR-204 is associated with an increased level of BDNF [24, 25]. Therefore, reducing the level of miR-204 in the PVN might lead to SNA via overexpressing BDNF.

Additionally, a study by Li DP and Pan HL showed enhanced glutamatergic synaptic input to PVN sympathetic neurons in hypertension [26]. In silico data showed that glutamate receptor (Grin2b) is a potential target for miR-204; therefore, new findings of glutamatergic synaptic plasticity in the PVN and miR-204 not only improve understanding of molecular mechanisms involved in the heightened activity of the SNS but also propose new therapeutic targets for treating drug-resistant neurogenic hypertension. Finally, accumulating evidence suggests that miR-204 downregulation in the hypothalamus could influence SNS and increase the SNA leading to hypertension.

SNA and cardiac electrophysiology

The autonomic nervous system (ANS) plays an essential role in regulating normal cardiac electrophysiology [27]. The sinoatrial (SA) node, the heart's natural pacemaker, consists of a cluster of cells situated in the upper part of the right atrium wall [28]. The SA node generates electrical impulses in the heart and is innervated by the SNS [29]. The ANS increases sympathetic outflow to the SA node in order to increase heart rate [29]. Alterations in autonomic tone may induce changes in local cellular electrophysiology that can manifest clinically as changes in heart rate and rhythm which eventually evolve into arrhythmia [29].

The parasympathetic division also innervates the SA node [30], and together, the sympathetic and parasympathetic divisions play a critical role in fine-tuning heart rate [31]. An increase in SNA is often associated with a decrease in parasympathetic nerve activity (PSNA) [32]. The vagus nerve is a PSN known to innervate the heart and regulate its rhythm [33, 34]. A chronic decrease in vagal nerve activity will lead to an uncontrolled increase in heart rate, creating a high risk for arrhythmia [35]. Chronic increased sympathetic activity, decreased parasympathetic activity and cardiac electrophysiology alteration are significant factors in developing hypertension [34].

Noradrenaline (NE) released by activating sympathetic nerve fibers to the SA node in the heart acts through the beta-adrenergic receptor 1 (β1) to increase SA node firing, thus increasing heart rate and contractility [29, 36]. As suggested above, a decrease in miR-204 can significantly increase the SNA leading to more NE secretion, which eventually may lead to an alteration of normal cardiac electrophysiology.

As well, the vagus nerve also innervates the SA node and acts on the firing rate leading to a slower heart rate. The parasympathetic division releases acetylcholine (Ach) that binds to the muscarinic receptor (M2) in the SA node [36]. The M2 receptor is a Gi-coupled protein that , once activated by Ach, will delay the firing of the SA node [29, 37-39]. 

Based on this evidence, it would be interesting to study the effect of hypothalamic miR-204 deletion on sympathetic and parasympathetic activity, heart electrophysiology, and hypertension. Hypothalamic miR-204 downregulation can increase the level of BDNF and Grin2b, leading to increased SNA and decreased PSNA. This, in turn, can induce heart electrophysiology damage eventually leading to hypertension. 

SNA and vasomotor function

ANS is also known to control the vasomotor activity of large arteries and small resistance arteries [40]. Literature has strongly established that sympathetic activity and vascular function are key factors in the development and prognosis of cardiovascular events and disease [40]. NE activates the alpha 1 (α1) adrenergic receptor, located in vascular smooth muscle cells (VSMC), to induce vasoconstriction [29, 41-43]. This, in turn, increases systemic vascular resistance and afterload [44]. The sympathetic nerves that innervate the vasculature display a physiological level of vascular tone activity. Increasing sympathetic outflow beyond this physiological level can cause more vasoconstriction that eventually leads to hypertension. Since we showed evidence that hypothalamic miR-204 downregulation leads to increased SNA, it would also be interesting to study the effect of this downregulation on vascular tone damage-induced hypertension.

Microbiota central miR-204 and cardiac electrophysiology

Commensal bacteria that inhabit the gut can modulate BP [45]. Interestingly, normotensive animals become hypertensive when transplanted with gut microbiota from hypertensive animals and vice versa [9]. Furthermore, patients with hypertension show specific alterations in gut microbial flora, suggesting that changes in gut microbial load and diversity (dysbiosis) contribute to the pathogenesis of hypertension [46-48]. Studies have shown that gut microbiota and its metabolites can play an important role in regulating cardiovascular diseases [49-51]. Recently, the potential role of the gut in the pathophysiology of arrhythmia and heart failure (HF) has received more attention [10, 52]. It has been shown that changing the composition of gut microbiota regulates the risk of HF [53, 54]. While more studies are needed to depict the mechanisms by which the microbiome regulates heart function, based on the previous evidence, we cannot overlook the fact that it also can affect the heart through the brain, thus leading to arrhythmia and hypertension.

**Figure 1:**
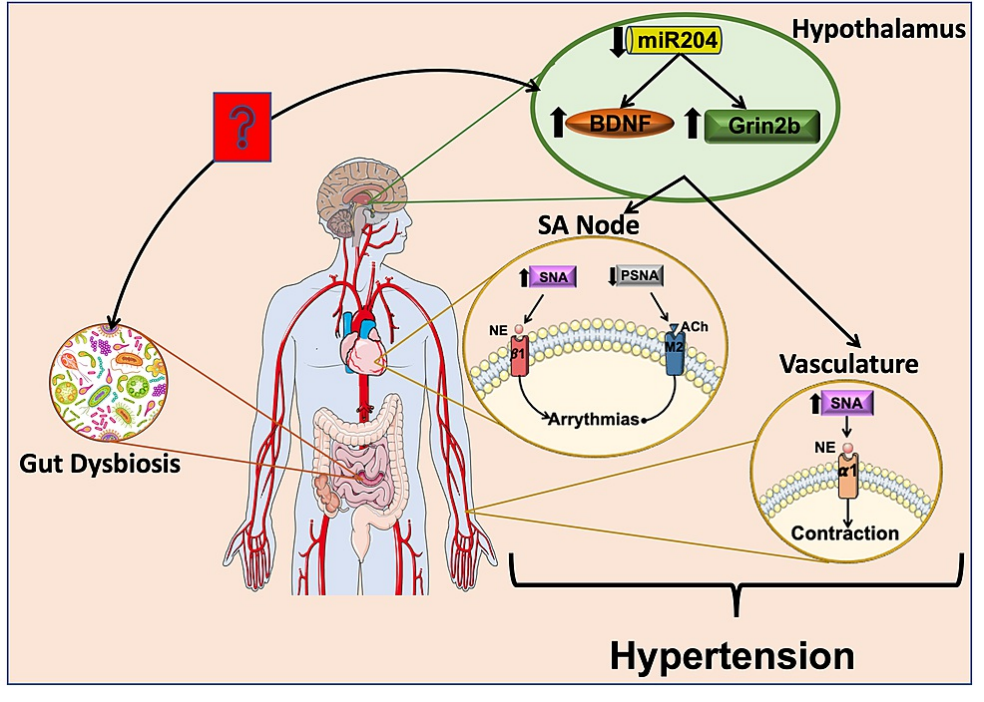
Mechanistic model. Gut dysbiosis decreases miR-204 level in the hypothalamus. Decreased miR-204 in the hypothalamus increases BDNF and Grin2B. These hypothalamic changes cause: 1) more SNA leading to pathophysiological levels of NE. NE acts on the ß1 receptor on the SA node and α1 receptor on the vasculature to produce cardiac and vessel contraction, respectively; and 2) less PSNA leading to a lower level of Ach. Ach activates the M1 receptor to reduce cardiac contraction. Deregulation in the level of NE and Ach will induce abnormalities in the heart and vessels, ultimately resulting in hypertension. BDNF: Brain-derived neurotrophic factor; Grin2b: Glutamate receptor 2b; SNA: Sympathetic nerve activity; PSNA: Parasympathetic nerve activity; NE: Noradrenaline; Ach: Acetylcholine; ß1: Beta 1 receptor; M1: Muscarinic receptor 1; α1: alpha 1 receptor; SA: sinoatrial node.

The gut microbiota is implicated in health and diseases through the regulation of host miRNAs [55]. In a previous study, we demonstrated that altering microbiota using antibiotics downregulated miR-204 in the vasculature [56]. Additionally, the absence of commensal bacteria resulted in the downregulation of miR-204 in the amygdala [15]. Altogether, these data indicate that microbiota can regulate miR-204 systematically as well as in the CNS, although the mechanism by which microbiota regulates miR-204 is yet to be determined. Yet, taken together, if the gut can affect hypothalamic miR-204, it will indeed affect the cardiovascular system function through the imbalance between sympathetic and parasympathetic nerve activity. The precise mechanisms on the above interactions and connections have not been fully elucidated and, thus, developing the proposed hypothesis in this review will open the door to a new concept of gut-brain axis miRNAs and cardiovascular diseases (Figure 1).

Future directions

More attention should also be paid to the fact that electric pulses are not only controlled by the SA node. The atrioventricular (AV) node is part of the heart’s electrical conduction system [57]. The signal generated by the SA node passes through the AV node to the ventricles, causing them to contract in order to pump blood into the circulatory system [58]. Disturbance anywhere along this electrical pathway can cause arrhythmia. Like the SA node, the AV node is innervated by both SNS and PSNS [59, 60]. In the AV node, conduction velocity is markedly increased by NE [61]. Chronic SNA can lead to sustained increases in conduction velocity and lead to arrhythmias. Parasympathetic activation manifested by Ach release from the vagal nerve decreases conduction velocity at the AV node by decreasing membrane action potential [29]. Since hypothalamic miR-204 might regulate the SNS and the PSNS, it would be interesting to study its downregulation effect on the AV node function as well. 

More studies should also investigate the microbiota effect on hypothalamic miR-204 and the Renin-Angiotensin-Aldosterone System (RAAS). Renin is synthesized in juxtaglomerular cells (JC) in the kidneys [62], and it is already known that renin secretion is regulated by NE release [63]. Decreased levels of hypothalamic miR-204 can lead to increased renal SNA. Therefore, more NE will bind to the ß1 receptor on the JC and lead to higher levels of renin secretion. Renin is the major determinant of the angiotensinogen II (AngII) production rate [64]. AngII is a key component in regulating BP [65]. Consequently, when renin is increased, AngII will also increase. A chronic increase in AngII will lead to hypertension and heart remodeling that might lead to arrhythmia and HF [66].

## Conclusions

Despite a wealth of studies over the last decade that has provided major insights into the gut-brain axis, miRNAs, and cardiovascular system control, we still have significant gaps in our knowledge. Thus, the central hypothesis of this review lies at the intersection of gut dysbiosis, miR-204, heart and vessels, and hypertension. It posits that gut dysbiosis, through reduction of hypothalamic miR-204, is a key driver of increased sympathetic overdrive to the heart and its vessels, leading to hypertension. It also puts forth the hypothesis that parasympathetic activity is reduced due to hypothalamic miR-204 reduction. Therefore, hypothalamic miR-204 is speculated to play a key role in a microbiota-gut-brain crosstalk and heart damage-induced hypertension.

The hypothesis discussed in this review is highly novel and, if accomplished, will lay the foundation for microRNA-based and/or microbiota-based therapeutics to prevent or treat heart damage-induced hypertension.
